# Redox-Stable Electrodes for Ethane Dehydrogenation Based on Proton Ceramic Electrochemical Reactors

**DOI:** 10.1021/acsaem.4c03281

**Published:** 2025-03-27

**Authors:** Elena Barrio-Querol, Laura Almar, David Catalán-Martínez, Kwati Leonard, José Manuel Serra, Sonia Escolástico

**Affiliations:** † Instituto de Tecnología Química (Universitat Politècnica de València-Consejo Superior de Investigaciones Científicas), València 46022, Spain; ‡ Center for Energy Systems Design (CESD), International Institute for Carbon-Neutral Energy Research (I2CNER), Kyushu University, 744 Motooka, Nishi-ku, Fukuoka 819-0395, Japan

**Keywords:** protonic electrochemical cells, redox-stable electrodes, H_2_ production, ethane dehydrogenation reaction (EDH), process intensification

## Abstract

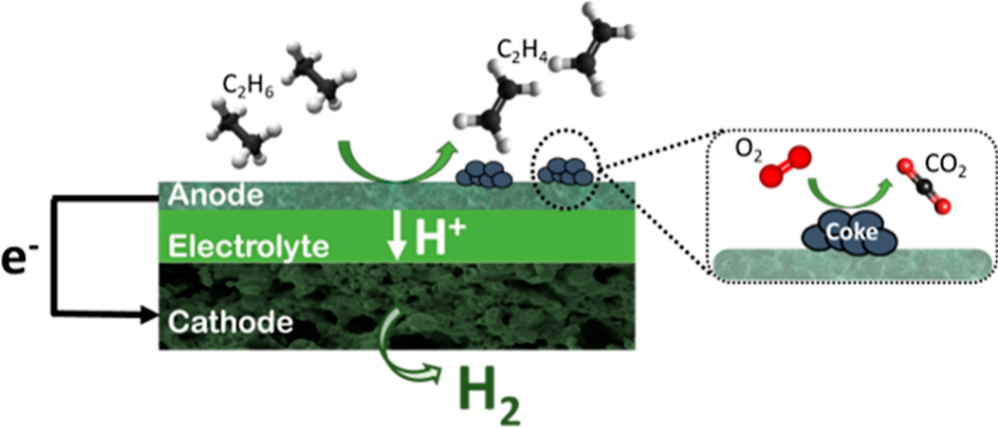

Ethylene is one of the most widely used components in the chemical industry, but the main manufacturing route involves significant energy consumption and generates substantial CO_2_ emissions. Proton ceramic electrochemical reactors (PCERs) offer great potential for process intensification and could play a key role in ethane dehydrogenation (EDH) by extracting H_2_ produced during the reaction. This process not only improves the reaction yield but also enables the production of a pure separated H_2_ stream. However, nonoxidative EDH reaction conditions lead to coke formation, which is further increased by H_2_ extraction, resulting in a decrease in system performance. Therefore, to successfully integrate PCER technology into ethylene production, it is crucial to develop stable redox electrodes that can withstand both nonoxidative H_2_ extraction and coke oxidation conditions. In this work, we study different composite electrodes based on the perovskite La_0.8_Sr_0.2_Cr_0.5_Mn_0.5_O_3−δ_ (LSCM) combined with the proton conductor BaCe_0.55_Zr_0.3_Y_0.15_O_3−δ_ (BCZY_5515_). The electrochemical performance was characterized by using electrochemical impedance spectroscopy under both oxidizing and reducing conditions. The data analysis indicates that surface processes limit electrode operation. The infiltration of Pt and CeO_2_ nanoparticles in the electrode enhanced the electrochemical performance, improving it by a factor of 10 at 700 °C. The optimal electrochemical performance was observed for the LSCMF/BCZY_5515_ (La_0.8_Sr_0.2_Cr_0.5_Mn_0.25_Fe_0.25_O_3−δ_/BaCe_0.55_Zr_0.3_Y_0.15_O_3−δ_) electrode infiltrated with Pt/CeO_2_, demonstrating promising properties as a redox-stable electrode. Finally, we evaluated the nonoxidative EDH reaction using a PCER based on a Ni–SrZr_0.5_Ce_0.4_Y_0.1_O_2.95_ (SZCY541) supported cell with a LSCMF/BCZY_5515_ anode infiltrated with Pt/CeO_2_ and a thin BaZr_0.44_Ce_0.36_Y_0.2_O_3−δ_ electrolyte.

## 1 Introduction

The demand for ethylene, a crucial component in the chemical industry, is projected to reach 260 million tons by 2035.^1^ The current manufacturing route, steam thermal cracking of ethane, is energy-intensive and results in significant CO_2_ emissions (1–2 tons).^2^ To tackle the challenges of ethylene production while reducing both costs and carbon emissions, proton-conducting electrochemical reactors [proton ceramic electrochemical reactors (PCERs)] are a promising alternative. These reactors integrate electrochemical H_2_ separation with catalytic reactions, allowing for the efficient use of electricity and facilitating the breaking of C–H bonds. By extracting H_2_ at the negative electrode, PCERs enhance the production of valuable chemicals and shift the equilibrium of the reaction, leading to increased product yields and significantly lower operating temperatures.^3,4^ Protons are transported across the membrane, allowing the separation, purification, and electrochemical compression of pure H_2_, contributing to a more sustainable and environmentally friendly production process.

Process intensification and pure on-site hydrogen production using electrochemical reactors for ethane dehydrogenation (EDH) have been highlighted in previous studies.^5−8^ For instance, Ding et al.^9^ demonstrated electrochemical dehydrogenation of ethane to ethylene using a protonic ceramic electrochemical cell (PCEC), reporting an ethylene selectivity of 100% at 400 °C; however, coking was observed at temperatures above 500 °C. The significant coke produced during EDH causes catalyst deactivation, which requires catalyst regeneration, usually under oxidizing conditions. A stable electrode is essential under both reducing and oxidizing conditions in the electrochemical reactor, presenting a major technical challenge.

Redox-stable electrodes are designed to function effectively in both oxidizing and reducing environments. When used with carbon fuels, these electrodes help minimize coke formation through controlled oxidation, promoting efficient combustion or carbon deposit reoxidation. To achieve this, the electrodes must possess good chemical and thermal stability along with high electronic conductivity under specific reaction conditions. However, only a limited number of studies on redox-stable electrodes for protonic ceramic electrochemical cells (PCECs) are available in the literature.^10−13^ Recently, an LSM/BCZY27 electrode infiltrated with the binary Pt/CeO_2_ has been proposed as a redox-stable electrode for CH_4_ conversion.^14^ However, investigations in reversible solid oxide electrochemical cells (ReSOECs) showed that LaMnO_3−δ_ perovskite exhibits redox instability at temperatures above 800 °C.^15,16^ For this reason, current research on stable electrodes under redox conditions is based on materials such as Mn-doped LaCrO_3−δ_ perovskites, which have high electronic conductivity and good stability in reducing atmospheres.^13−18^ Tao et al. used La_0.75_Sr_0.25_Cr_0.5_Mn_0.5_O_3−δ_ (LSCM) as a promising alternative redox electrode material for SOFCs, showing a stable phase structure under reducing atmospheres and favorable electrocatalytic activity for methane and hydrogen oxidation.^17^

Building upon prior research, this study aims to explore different composite electrodes capable of serving as redox-stable electrodes for EDH reactions in proton-conducting electrochemical cells. We develop redox-stable electrodes based on the material La_0.8_Sr_0.2_Cr_0.5_Mn_0.5_O_3−δ_, aiming to improve their electrochemical properties and stability under both oxidizing and reducing conditions. For this purpose, we investigate the influence of partial substitution of trivalent elements in the A and B positions, leading to increased oxygen vacancies and defects in the perovskite structure of the reference material, thus enhancing their electrochemical properties and stability.^18^ Furthermore, we assess the compatibility and electrochemical performance of candidate electrodes with the protonic material BaCe_0.55_Zr_0.3_Y_0.15_O_3−δ_ (BCZY_5515_) under redox conditions. We also optimize the electrode exhibiting the best characteristics and explore its potential application as a redox electrode in EDH. Lastly, the nonoxidative EDH reaction was performed using a protonic electrochemical cell, with the best-developed electrode as an anode.

## 2 Experimental Section

The developed redox-stable electrode candidates are formed by the protonic conducting material BaCe_0.55_Zr_0.3_Y_0.15_O_3−δ_ (BCZY_5515_) and different perovskite materials as electronic phases. The electronic phases selected were La_0.8_Sr_0.2_Cr_0.25_Mn_0.75_O_3−δ_ (LSCM_75_), La_0.7_Sr_0.1_Ce_0.2_Cr_0.5_Mn_0.5_O_3−δ_ (LSCeCM), La_0.7_Sr_0.1_Nd_0.2_Cr_0.5_Mn_0.5_O_3−δ_ (LSNCM), La_0.8_Sr_0.2_Cr_0.5_Fe_0.5_O_3−δ_ (LSCF), and La_0.8_Sr_0.2_Cr_0.5_Mn_0.25_Fe_0.25_O_3−δ_ (LSCMF). All of these materials were synthesized by complexation–gelation processes, followed by the final controlled pyrolysis. The nitrate precursors (La(NO_3_)_3_ 6H_2_O (99.9%, Sigma-Aldrich), Nd(NO_3_)_3_ 6H_2_O (99.9%, Sigma-Aldrich), Sr(NO_3_)_2_ (99%, Sigma-Aldrich), Cr(NO_3_)_3_ 9H_2_O (99%, Sigma-Aldrich), Fe(NO_3_)_3_ 9H_2_O (98%, Sigma-Aldrich), and Mn(NO_3_)_2_ 4H_2_O (98%, Alfa Aesar)) were dissolved in distilled water with an appropriate amount of ethylene glycol (99.8%, anhydrous, Thermo scientific) and citric acid (99%, Alfa Aesar). The solution was heated to 220 °C to accelerate the formation of polyester through the reaction of citric acid and ethylene glycol, resulting in the gelation of the reaction mixture and subsequent foaming. The obtained powder was calcined at 1150 °C for 7 h to form the crystalline phase.

The BaCe_0.55_Zr_0.3_Y_0.15_O_3−δ_ material was synthesized by using the solid-state reaction method. All the precursors (ZnO_2_ (Alfa Aesar, 99.5%), Y_2_O_3_ (Alfa Aesar, 99.9%), CeO_2_ (Alfa Aesar, 99.9%), and BaCO_3_ (Alfa Aesar, 99–101%)) were milled for 48 h and then calcined at 1500 °C for 7 h.

X-ray diffraction (XRD) was used to perform structural characterization of the synthesized materials to ensure the formation of the appropriate single phase. XRD measurements were carried out using a CubiX FAST equipment with CuKα1,2 radiation and an X’Celerator detector in Bragg–Brentano geometry, covering the 2θ range from 20 to 70°. The obtained XRD patterns were analyzed using X’Pert Highscore Plus software (PANalytical).

To study the chemical compatibility of the selected electronic phases with BCZY_5515_, XRD measurements were performed after a heat treatment at 1050 °C for 7 h. Before the calcination step, the powders were mixed by combining 50% v/v of the electronic and protonic phases.

Symmetrical cells were fabricated by using screen printing for the electrode deposition. Inks were prepared by mixing 50% v/v of each phase (BCZY_5515_ as the proton phase and LSCM_75_, LSCeCM, LSCMF, LSCF, or LNSCM as the electronic phase) with terpineol (90%, Sigma-Aldrich) and ethyl cellulose (Sigma-Aldrich) in a three-roll mill. The electrode inks were painted on both sides of the BCZY_5515_ dense electrolyte with an active area of 0.64 cm^2^. The sintering temperature of the electrodes was set at 1050 °C for 7 h. To ensure proper current collection, gold paste was applied as a mesh on top of the sintered electrodes.

To enhance the catalytic activity and, subsequently, the electrochemical performance of the electrodes, LSCMF-, LSCF-, and LNSCM-based electrodes were infiltrated with Pt/CeO_2_ precursors.^14^ Two solutions were prepared by using ethanol and water: a 0.15 M solution of (Pt(NH_3_)_4_)(NO_3_)_2_ (99.995%, Sigma-Aldrich) and a 2 M solution of Ce(NO_3_)_3_·6H_2_O (99.999%, Sigma-Aldrich). The Pt solution was first dropped over the entire electrode surface and calcined at 800 °C. Next, the Ce solution was added and calcined again at 800 °C.

The electrochemical performance of the manufactured cells was evaluated by using electrochemical impedance spectroscopy (EIS) with a Solartron Analytical 1470E CellTest System frequency response analyzer in the 10^–2^ to 10^6^ Hz frequency range. The collected impedance data were analyzed and fitted using the ZView software. The sintered cells were placed in a quartz reactor between Pt meshes and tested in the temperature range between 800 and 600 °C under oxidizing and reducing conditions (wet air and wet 10% H_2_ in Ar). For the best-developed performing electrode, the stability was evaluated upon redox cycling by switching the operation atmosphere between synthetic air and 10% H_2_–10% C_2_H_6_ in Ar, both saturated in 3%H_2_O, trying to resemble the reaction conditions and the catalyst regeneration step.

BaZr_0.44_Ce_0.36_Y_0.2_O_3−δ_ electrode-supported cells were fabricated by a sequential tape casting route, using a KARO cast 300-7 microtape casting device (KMS Automation GmbH Germany). The electrode cement slurry consisted of commercial NiO powder (Vogler, raw material) and SrZr_0.5_Ce_0.4_Y_0.1_O_2.95_ (SZCY541) in a 60:40 weight ratio along with an ethanol and methyl ethyl ketone (34:66) mixture. Nuosperse FX9086 served as the dispersing agent. In addition, PVB-98, Solusolv 2075, and polyethylene glycol (PEG) 400 were added as plasticizers and binders, respectively. The mixture was homogenized in a Thinky vacuum mixer and rested for 48 h to deair and completely dissolve the binder before casting. The electrolyte slurry was prepared using a previously reported procedure^19,20^ and cast onto a silicone-coated polymeric (polyethylene terephthalate) foil. After proper drying at room temperature, the NiO-SZCY541 slurry, one without and with a pore former, was cast directly on top of the electrolyte layer, successively with a 6 h drying interval. This method ensures defect-free electrolyte layers due to the high surface quality of the foil. The green tapes were then precisely cut to the desired dimensions and sintered at 1350 °C for 5 h to obtain an integrated BaZr_0.44_Ce_0.36_Y_0.2_O_3−δ_/NiO-SZCY541 composite ceramic referred to as half-cell.

The nonoxidative EDH reaction was performed in a double-chamber quartz reactor, and the cell sealing was obtained using an Ag-based gasket. Pt present in the anode, as well as in the current collector, acts as a catalyst for the reaction. Before the reaction test, the cathode (NiO-SZCY541 electrode) was reduced at 700 °C, employing an atmosphere of 20 mL·min^–1^ of H_2_ and 80 mL·min^–1^ of Ar. The electrochemical properties of the cell were studied by *I*–*V* curves and EIS measurements. Then, H_2_ pumping through the cell was evaluated at 650 °C, feeding 5 mL·min^–1^ of H_2_ and 95 mL·min^–1^ of Ar in the anode chamber and humidified Ar (80 mL·min^–1^) in the cathode chamber. H_2_ pumping conditions were selected to mimic the H_2_ concentration generated during the EDH reaction. Finally, the EDH reaction was carried out at 650 °C feeding a gas stream composed of 20 mL·min^–1^ of C_2_H_6_ and 30 mL·min^–1^ of He in the anode chamber, whereas 80 mL·min^–1^ of humidified Ar (3%H_2_O) was fed in the cathode chamber. The gas outlet of the reaction was analyzed using a micro-GC 990 (Agilent) gas chromatograph equipped with Molsieve 5A and PoraPlot-*Q* glass capillary modules. EDH reaction was carried out in 4 different steps: (a) 60 min without applying current, (b) 20 min applying 300 mA·cm^–2^, (c) current was not applied for 1180 min, and finally, (d) 300 mA·cm^–2^ was applied for 100 min. Then, a mixture consisting of 20% air in Ar was fed into the anode to remove the coke formed, and the electrochemical performance of the cell was evaluated to verify the stability of the electrode.

The microstructures of the electrodes and the electrode–electrolyte interface were investigated with a Zeiss Ultra 55 field-emission scanning electron microscopy. Raman spectroscopy measurements were performed with a Renishaw Raman spectrometer using a 785 nm laser equipped with an Olympus microscope and a CCD detector at room temperature.

## 3 Results

### 3.1 Chemical Compatibility Study of the Composite Materials

Five different materials based on the La_1–*x*_Sr_*x*_Cr_0.5_Mn_0.5_O_3_ material were selected as electronic material candidates, and BaCe_0.55_Zr_0.3_Y_0.15_O_3−δ_ was selected as the protonic phase for the developed composite electrodes. The parent composition, La_0.8_Sr_0.2_Cr_0.5_Mn_0.5_O_3_ (LSCM), was selected based on a previous work in which the potential of the material as a redox-stable electrode was demonstrated.^14^ In the present study, the following strategy was employed to boost the performance of this material by improving electronic conductivity, electrocatalytic activity, and redox stability: (i) doping the A-site of LSCM with different lanthanides,^21^ (ii) increasing the Mn concentration, and (iii) fully or partially substituting Mn with Fe.^22^

The stoichiometry of the individual compounds and the nomenclature used are listed in Table 1.

**Table 1:** Stoichiometry of the Studied Individual Compounds and the Nomenclature Used

individual compounds	nomenclature
La_0.8_Sr_0.2_Cr_0.25_Mn_0.75_O_3−δ_	LSCM_75_
La_0.7_Sr_0.1_Ce_0.2_Cr_0.5_Mn_0.5_O_3−δ_	LSCeCM
La_0.7_Sr_0.1_Nd_0.2_Cr_0.5_Mn_0.5_O_3−δ_	LSNCM
La_0.8_Sr_0.2_Cr_0.5_Fe_0.5_O_3−δ_	LSCF
La_0.8_Sr_0.2_Cr_0.5_Mn_0.25_Fe_0.25_O_3−δ_	LSCMF
BaCe_0.55_Zr_0.3_Y_0.15_O_3−δ_	BCZY_5515_

**Figure 1:**
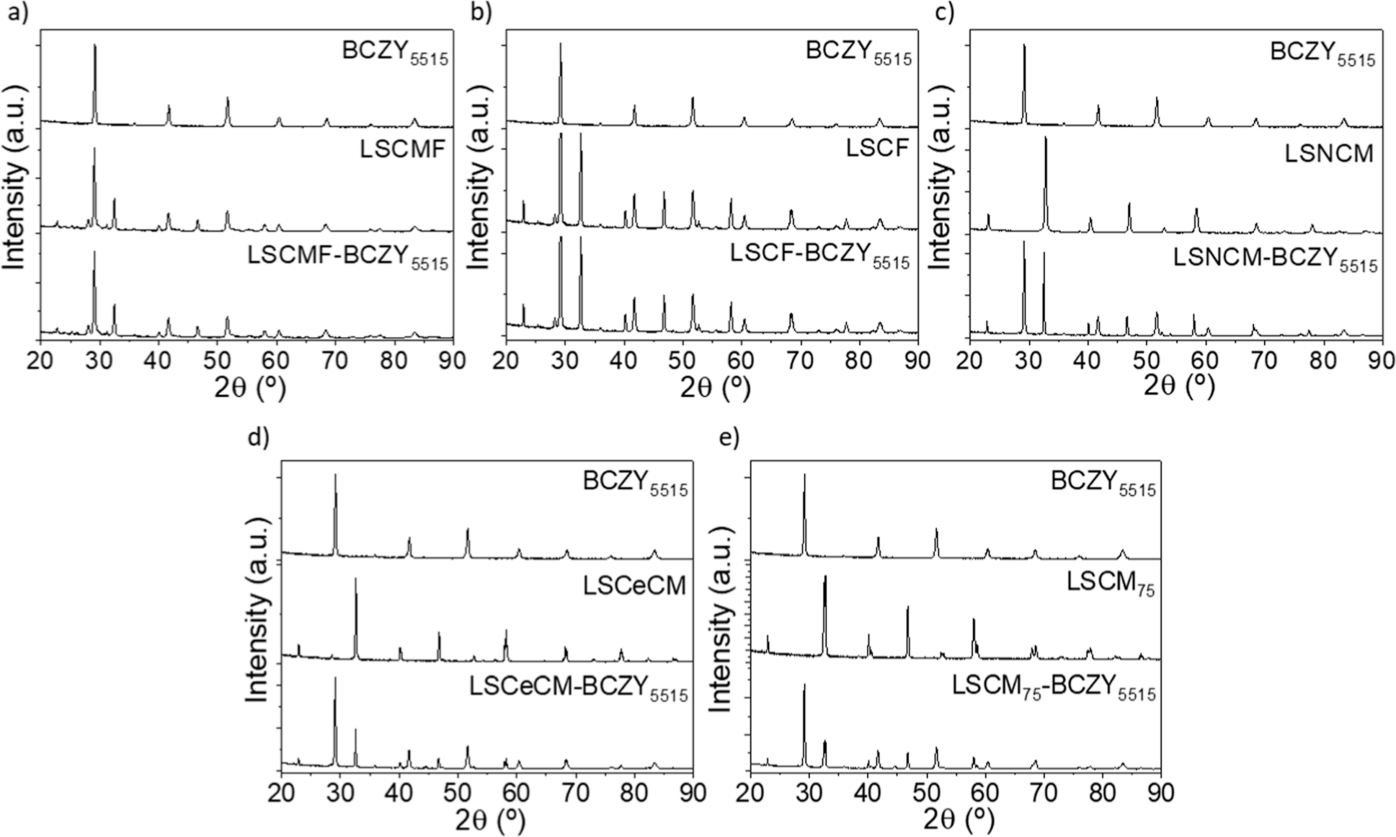
XRD patterns of powder mixing composites (a) LSCMF-BZCY_5515_, (b) LSCF-BZCY_5515_, (c) LSNCM-BCZY_5515_, (d) LSCeCM-BCZY_5515_, and (e) LSCM_75_-BCZY_5515_ after compatibility tests at 1050 °C for 7 h.

Figure 1 shows the XRD patterns of the individual phases and synthesized composite materials. The electronic components reveal a perovskite structure characterized by orthorhombic symmetry at room temperature, whereas the protonic material presents cubic symmetry. The chemical compatibility of the selected electronic phases with the proton phase (50% v/v of electronic and proton phases) was studied after a heat treatment at 1050 °C for 7 h in air. The XRD analysis reveals that all of the evaluated electronic materials are compatible with the selected BCZY_5515_ protonic phase since both phases can be observed in all samples without detecting any secondary phases.

### 3.2 Electrochemical Characterization of the Redox-Stable Electrodes

**Figure 2:**
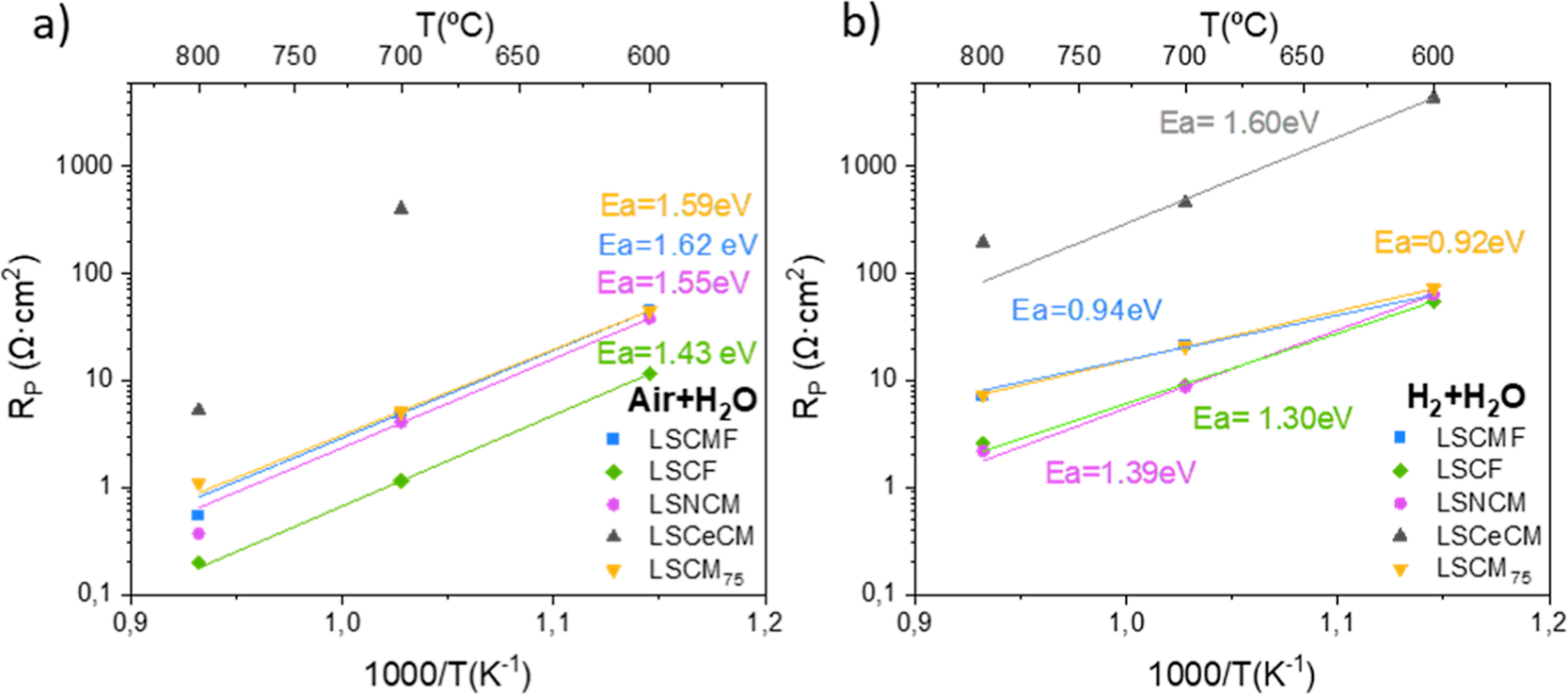
Polarization resistance (*R*_p_) as a function of temperature for the electrodes measured in symmetrical cells under humidified atmospheres composed of (a) air and (b) 10% H_2_ in Ar.

**Figure 3:**
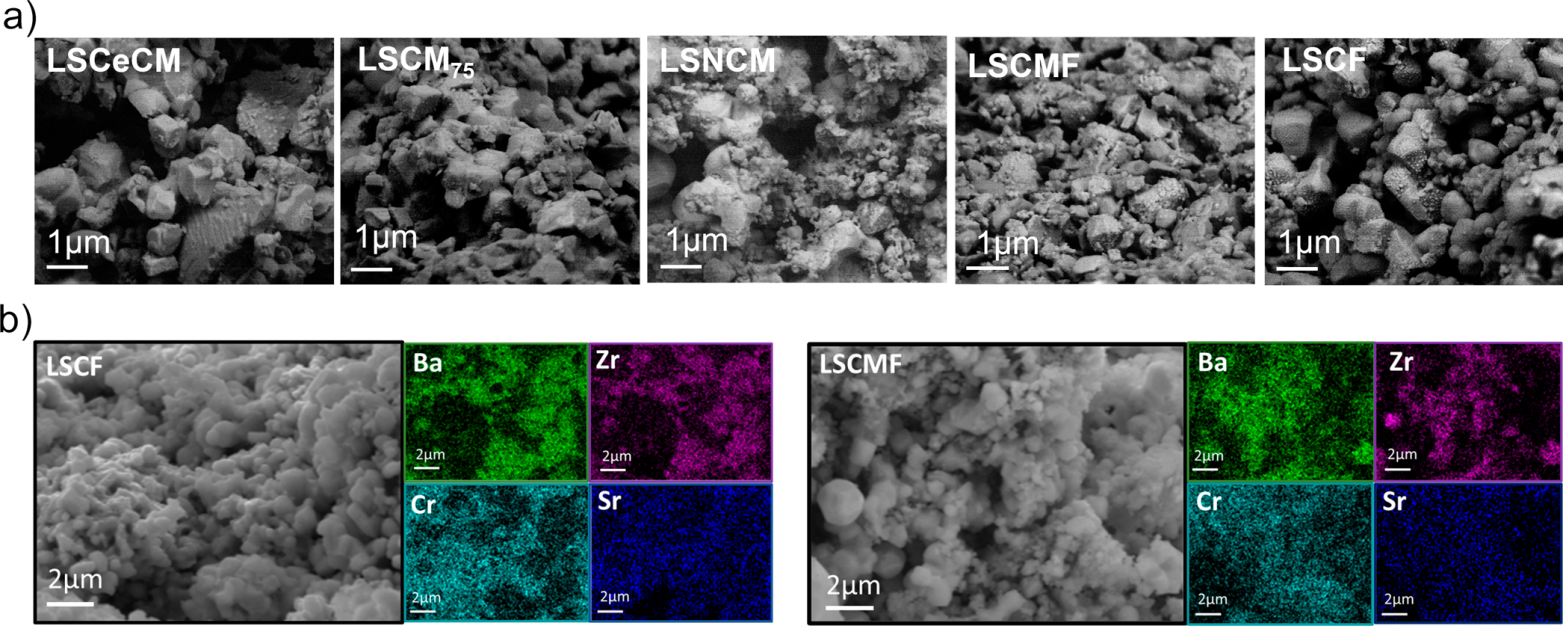
Microstructure of the electrodes: (a) BDS cross-section images of the composite electrodes after the electrochemical measurements and (b) EDS of the composites LSCF/BCZY_5515_ and LSCMF/BCZY_5515_ after the electrochemical measurements (b).

The electrochemical performance of the developed electrodes was evaluated by EIS using symmetrical cells under both oxidizing (humidified synthetic air, 3%H_2_O) and reducing conditions (humidified 10% H_2_ in Ar, 3%H_2_O) in the temperature range from 800 to 600 °C. Figure 2a,b shows the total polarization resistance (*R*_p_) obtained by fitting the EIS spectra as a function of the reciprocal temperature obtained for the different electrodes developed under oxidizing and reducing conditions. The best-performing electrode in both atmospheres is LSCF/BCZY_5515_, exhibiting a polarization resistance at 700 °C of 1.19 and 9.28 Ω·cm^2^, in oxidizing and reducing conditions, respectively. The electrodes LSNCM/BCZY_5515_, LSCMF/BCZY_5515_, and LSCM_75_/BCZY_5515_ showed similar performance under oxidizing atmospheres, with *R*_p_ values at 700 °C of 3.88, 4.99, and 5.09 Ω·cm^2^, respectively. On the other hand, under reducing conditions, LSNCM/BCZY_5515_ and LSCF/BCZY_5515_ showed the lowest *R*_p_, followed by LSCMF/BCZY_5515_ and LSCM_75_/BCZY_5515_, which also behaved similarly. The highest *R*_p_ was obtained for LCeSCM/BCZY_5515_ in both atmospheres. Regarding the activation energy (*E*_a_), the values obtained ranged between 0.98 and 1.66 eV and between 0.86 and 1.47 eV in oxidizing and reducing atmospheres, respectively. These *E*_a_ values are associated with limiting processes at low frequencies (*E*_a_ ≈ 1.17 eV),^23^ indicating that surface processes hindered by low oxygen activation and reduced electrocatalytic activity limit the performance of the developed electrodes. In summary, the materials studied show similar performance, with the exception of LCeSCM/BCZY_5515_, which exhibits a significantly lower performance compared to the other materials, possibly due to its coarser microstructure (see Figure 3). It is important to consider that the performance of an electrode depends on multiple factors, such as the electrocatalytic activity of the materials, surface area, porosity, electronic and ionic conductivities, and electrode–surface interaction. Therefore, variation in any of these properties can influence the overall polarization resistance values.

Figure 3a shows the cross-sectional images, using a backscattered electron detector (BDS), of the electrodes after electrochemical measurements, demonstrating a similar morphology with particle sizes ranging from 0.7 to 1 μm and good interconnection between the two phases. The LSCeCM electrode, however, exhibits larger particles, with the size exceeding 1 μm, which is likely associated with its higher polarization resistance. To further evaluate the elemental composition of the electrodes, LSCF/BCZY_5515_ and LSCFM/BCZY_5515_ electrodes were characterized using energy-dispersive X-ray spectroscopy (EDS) (Figure 3b). The mappings show good interconnection between the two phases, i.e., the proton conducting phase (BCZY_5515_) through Ba and Zr signals and the electronic phase (LSCF and LSCMF) through Cr and Sr signals.

Impedance spectra were fitted by using the equivalent circuit shown in the inset of Figure S1. The polarization resistance is calculated by summing the three polarization resistance contributions (*R*_P_ = *R*_HF_ + *R*_MF_ + *R*_LF_), where HF, MF, and LF refer to the high, medium, and low frequencies, respectively. Figure S1 shows the impedance spectra (Nyquist and Bode) recorded at 600 °C in wet air for the LSCMF/BCZY_5515_ electrode. In this spectrum, three distinct contributions can be identified: (i) LF contribution with associated frequencies of 10^–2^ to 1 Hz and capacitances of 10^–1^ to 10^–2^ F/cm^2^; (ii) MF contribution with associated frequencies of 10 to 100 Hz and capacitances between 10^–3^ and 10^–4^ F/cm^2^; and (iii) HF contribution with associated frequencies above 1 kHz and capacitances between 10^–5^ and 10^–7^ F/cm^2^. The LF and MF contributions are typically associated with elemental reactions occurring at the electrode surface, such as the adsorption and dissociation of gas molecules, which are part of the surface exchange kinetics. In contrast, the HF contribution is attributed to proton and electronic losses occurring at the interfaces between the electrode and the electrolyte or the electrode and the current collector.^24^

**Figure 4:**
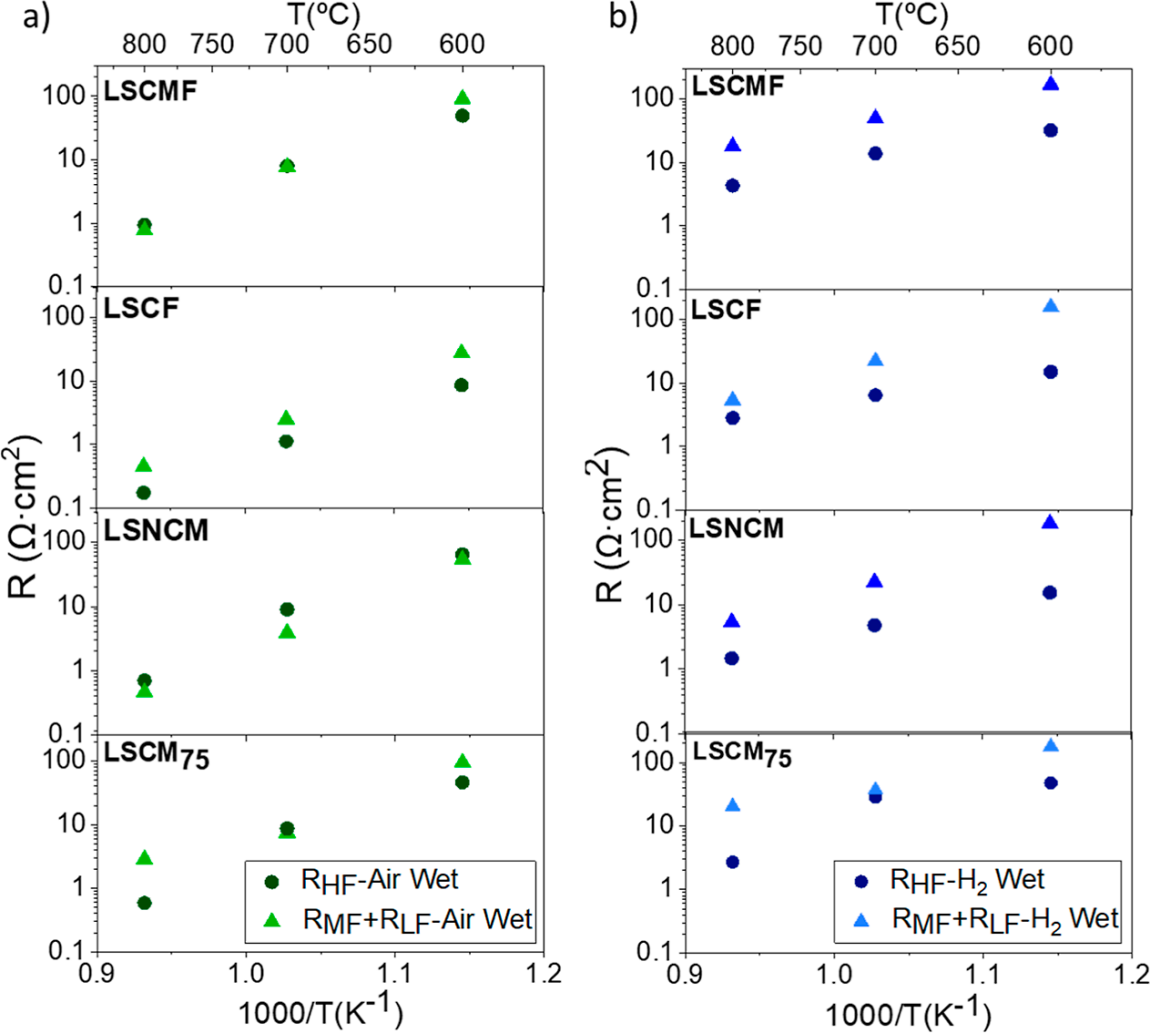
Resistance contributions at LF, MF, and HF of the symmetrical cells as a function of temperature under wet synthetic (a) air and (b) 10% H_2_ in Ar.

Figure 4 shows the different resistance contributions (*R*_HF_, *R*_MF_, and *R*_LF_) obtained from the EIS spectra fitting and deconvolution for the electrodes LSCMF/BCZY_5515_, LSCF/BCZY_5515_, LSNCM/BCZY_5515_, and LSCM_75_/BCZY_5515_ measured over the temperature range of 800–600 °C in humidified (3%H_2_O) synthetic air and 10% H_2_ in Ar. The fitted parameters can be found in Tables S1–S5.

Under oxidizing conditions, the electrodes show no important differences between high- and medium–low-frequency contributions. On the contrary, the medium–low frequencies become the main rate-limiting step in reducing atmospheres. This indicates that the electrodes are mainly limited by the elemental reactions occurring at the electrode surface, in line with the *E*_a_ values presented in Figure 2b.

### 3.3 Electrode Optimization through Nanoparticle Infiltration

The surface kinetics of electrodes in protonic conducting cells has been previously enhanced through the infiltration of Pt and CeO_2_ nanoparticles, obtaining the best results with the binary infiltration Pt/CeO_2_.^14^ Therefore, in this study, nanoparticles of Pt and CeO_2_ were infiltrated into the best-performing electrodes, i.e., LSCMF, LSCF, and LSNCM, to enhance the catalytic activity and boost the overall electrode performance.

**Figure 5:**
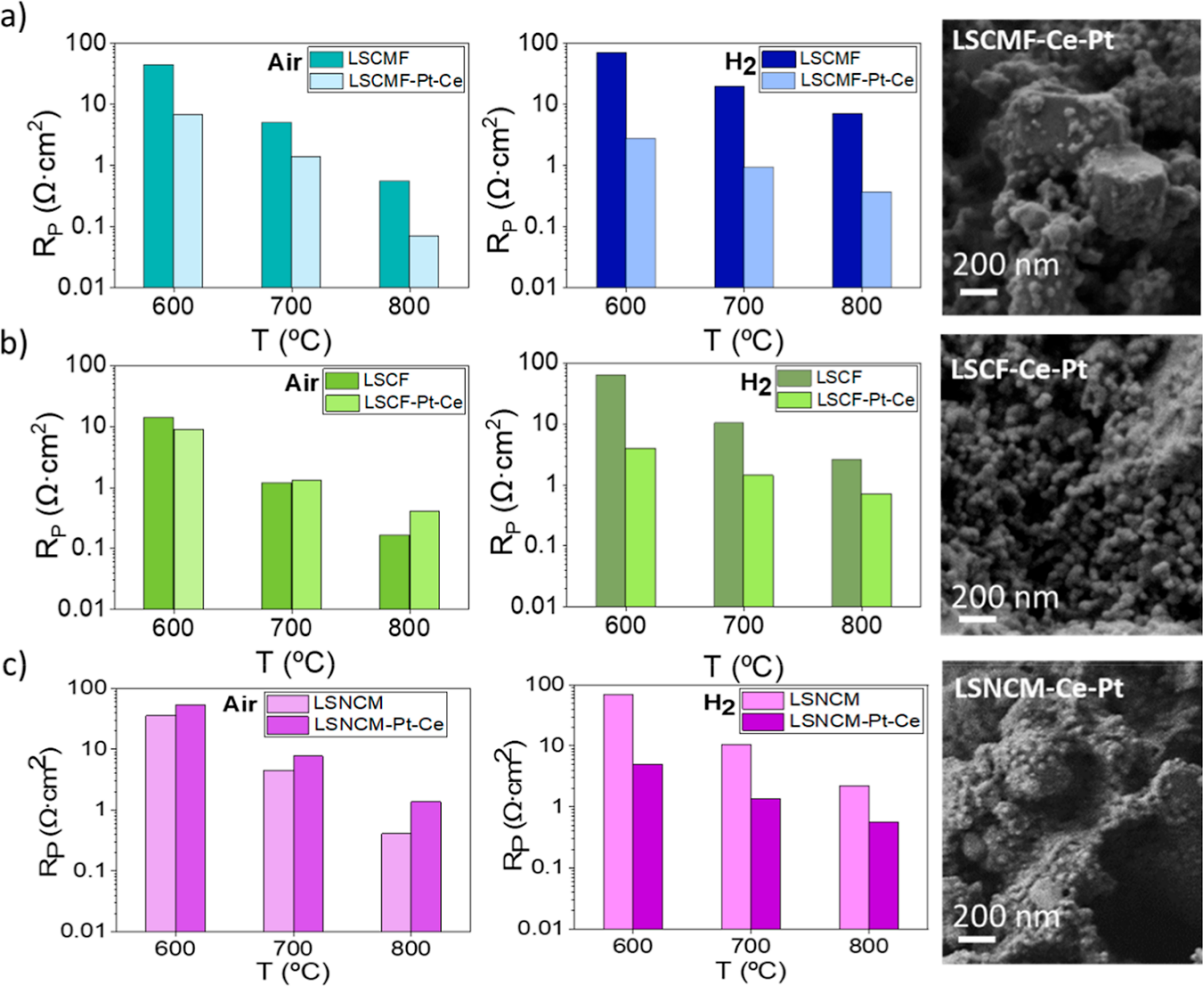
Polarization resistance as a function of temperature and SEM cross-sectional images for the composite electrodes (a) LSCMF/BCZY_5515_, (b) LSCF/BCZY_5515_, and (c) LSNCM/BCZY_5515_ infiltrated with Pt/CeO_2_ under humidified synthetic air and 10% H_2_ in Ar (3% of H_2_O).

Figure 5 compares the total polarization resistance for noninfiltrated electrodes and those infiltrated with Pt/CeO_2_, as studied by EIS at temperatures ranging from 800 to 500 °C under synthetic air and 10% H_2_ in Ar, both atmospheres humidified with 3%H_2_O (the fitted parameters can be found in Tables S6–S8). Under reducing atmospheres, the polarization resistance decreases by approximately 1 order of magnitude when the electrodes are infiltrated with binary Pt/CeO_2_ nanoparticles. On the other hand, improvements in oxidizing atmospheres are only observed for the LSCMF/BCZY_5515_ electrode. SEM cross-sectional images of the electrodes LSCMF/BCZY_5515_, LSCF/BCZY_5515_, and LSNCM/BCZY_5515_ infiltrated with Pt/CeO_2_ after the electrochemical measurements are shown in Figure 5a–c, respectively. The Pt/CeO_2_ nanoparticles exhibited a rounded morphology with sizes ranging from 10 to 30 nm and were uniformly dispersed across the porous electrode surfaces.

Figure S2 presents a comparative analysis of the EIS spectra (Nyquist and Bode plots) recorded at 700 °C in humidified air (a) and 10% H_2_ in Ar (b) between the electrode LSCMF/BCZY_5515_ with and without Pt/CeO_2_ infiltration. An important performance increase after infiltration is clearly observed. Specifically, LF and MF contributions (Table S6) decreased, which may be correlated with the increase in the number of active sites available, and this can be further attributed to the uniform dispersion of nanoparticles over the entire electrode surface, which exhibit superior intrinsic electrocatalytic activity. In fact, the infiltration of Pt and CeO_2_ nanoparticles, both catalytically active for the dissociation/association of H_2_,^25^ expands the triple-phase boundary (TPB), thereby increasing the number of active reaction sites.^26^ This infiltration introduces different types of TPBs: Pt predominantly contributes to the electronic phase of the TPB, while CeO_2_ supports both the electronic and ionic phases of the TPBs.^27^ Furthermore, a synergistic effect between CeO_2_ and Pt has been previously reported, enhancing the catalytic activity of CeO_2_, likely due to the strong electronic interaction between the two materials.^28^ Additionally, the HF contribution in the Bode plot (Figure S2) decreases,^29^ suggesting a potential interaction between the infiltrated nanoparticles and the electrode–electrolyte interface. This effect may be attributed to the oxygen vacancies in CeO_2_, which could enhance the ionic conductivity.

**Figure 6:**
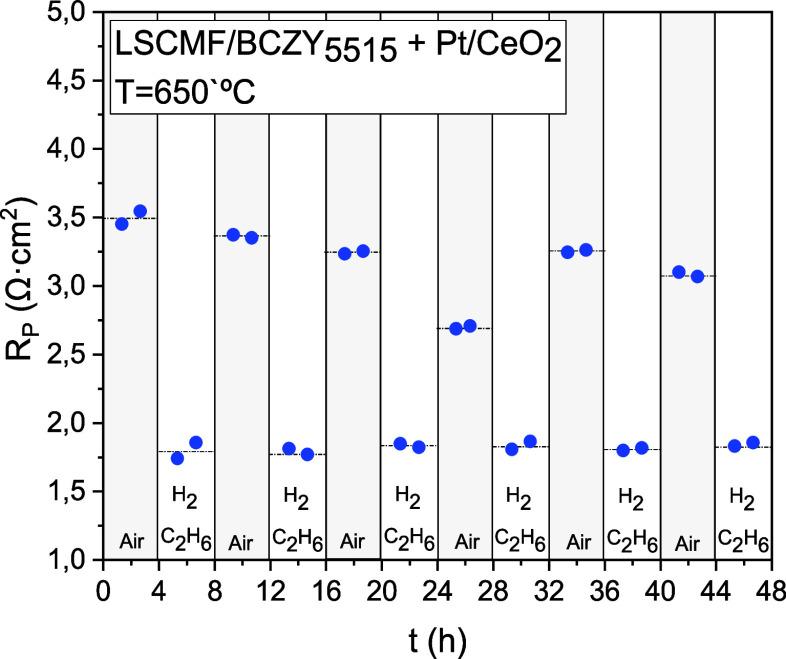
Redox stability cycles (humidified synthetic air-humidified 10% H_2_–10% C_2_H_6_ in Ar) at 650 °C for the composite electrode LSCMF/BCZY_5515_ infiltrated with Pt/CeO_2_.

The LSCMF/BCZY_5515_ electrode infiltrated with Pt/CeO_2_ was selected as the most promising redox-stable electrode developed in this work. In order to demonstrate its stability under EDH conditions and the subsequent regeneration steps, a new cell was exposed to consecutive redox cycles. The cycles were made by changing the atmosphere from synthetic air to 10% H_2_–10% C_2_H_6_ in Ar (both atmospheres humidified with 3%H_2_O) at 650 °C. The total polarization resistance values obtained from the EIS spectra measured during six consecutive redox cycles are shown in Figure 6. Polarization resistance remains stable with a value of 1.8 Ω·cm^2^ in reducing atmospheres after all of the redox cycles, indicating proper stability and reversibility of the material.

### 3.4 Cell Performance in the EDH Reaction

**Figure 7:**
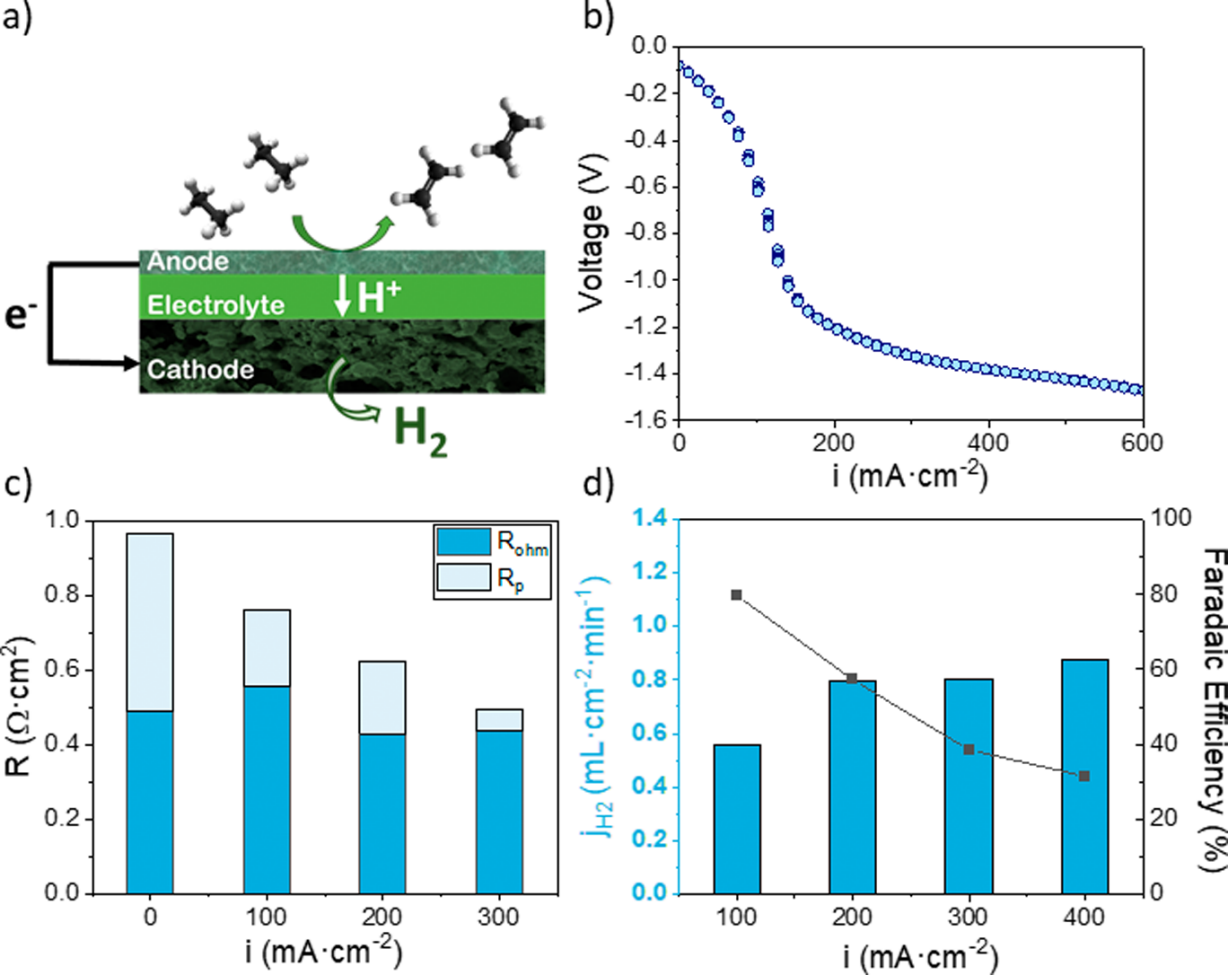
(a) Nonoxidative dehydrogenation of ethane processes by in situ H_2_ extraction with a proton-conducting electrochemical reactor (PCER). (b) *I*–*V* curve, (c) ohmic and polarization resistances extracted from EIS measurements, and (d) H_2_ pumping flow and the Faradaic efficiency as a function of the applied current density at 650 °C. In all measurements, a humidified atmosphere of 5% H_2_ in Ar was fed to the anode, and an Ar atmosphere was fed to the cathode chamber.

The nonoxidative dehydrogenation of ethane was performed using an electrochemical cell consisting of (a) an anode made of LSCMF/BCZY_5515_ infiltrated with Pt/CeO_2_ and (b) a 10 μm thick BaZr_0.44_Ce_0.36_Y_0.2_O_3−δ_ electrolyte supported on (c) a Ni-SZCY541 support acting as a cathode. A schematic of the cell is shown in Figure 7a. First, the electrochemical properties of the cell were characterized by *I*–*V* curves and EIS measurements, and H_2_ extraction was evaluated. These measurements were performed at 650 °C and fed with wet 5% H_2_ in Ar in the anode chamber and Ar in the cathode chamber. The cell shows good electrochemical performance, reaching 600 mA·cm^–2^ at 1.4 V, as evidenced by the *I*–*V* curve (Figure 7b) and the sum of ohmic and polarization resistances varying from 0.96 to 0.49 Ω·cm^2^ for 0–300 mA·cm^–2^ (Figure 7c). The polarization resistance decreases with the current density, while the ohmic resistance remains almost constant. H_2_ pumping was evaluated at different current densities (Figure 7d) without feeding C_2_H_6_. An H_2_ flux of 0.877 mL·min^–1^·cm^–2^ was obtained at the highest applied current density (400 mA·cm^–2^), and the Faradaic efficiency ranged from 79.7 to 31.5%, decreasing with the current density.

**Figure 8:**
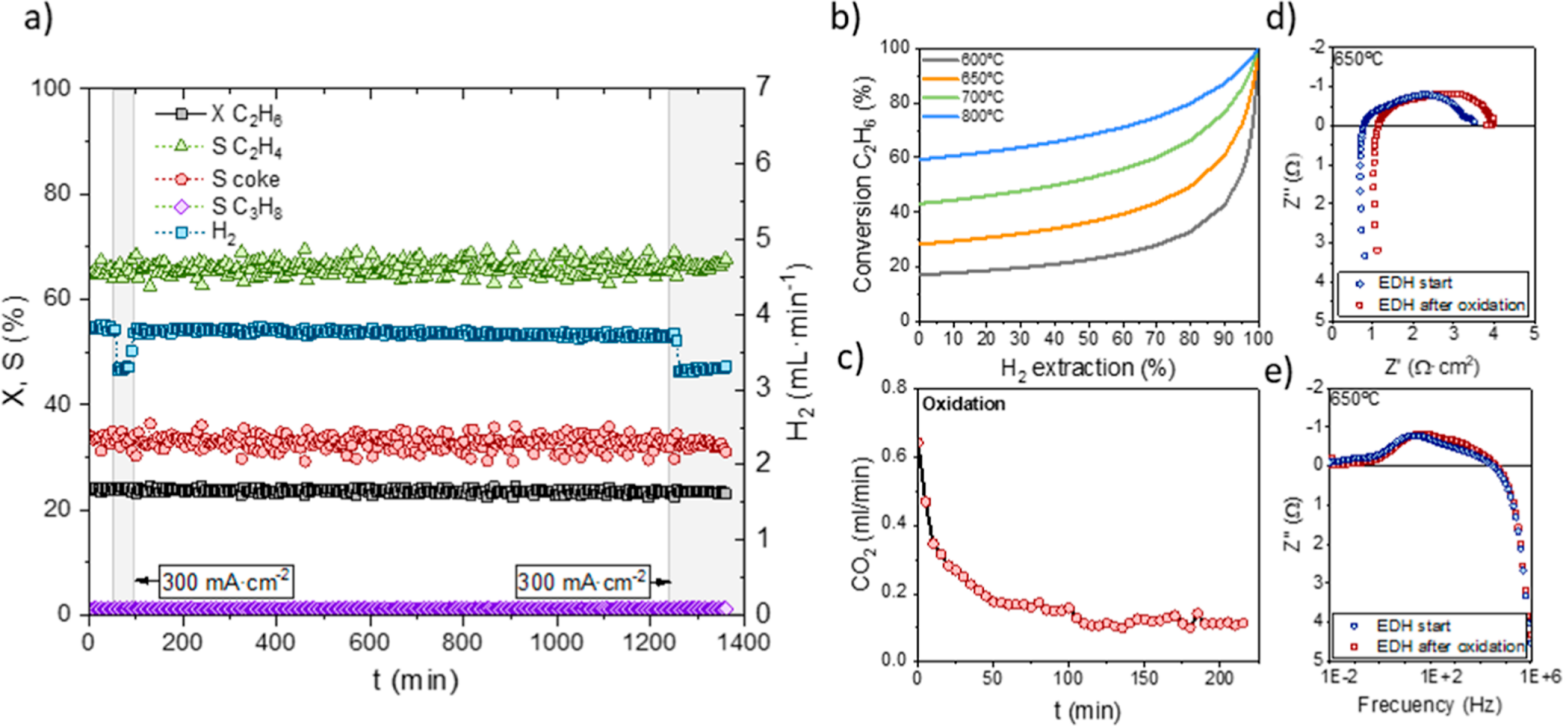
(a) Ethane conversion, product selectivity, and H_2_ flow extracted as a function of time and the applied current. (b) Effect of H_2_ extraction and temperature on equilibrium-limited ethane conversion (equilibrium calculated considering only ethylene and coke as reaction products). (c) CO_2_ production in the oxidation step in 20% air (Ar balance) after the EDH reaction. Comparison of Nyquist (d) and Bode (e) plots for the cell at the start of the reaction and after the oxidation step.

Then, the EDH reaction was evaluated at 650 °C, feeding a gas stream composed of 20 mL·min^–1^ of C_2_H_6_ and 30 mL·min^–1^ of He in the anode chamber (reaction chamber) and 80 mL·min^–1^ of humidified Ar in the cathode. The electrochemical properties of the cell under the reaction conditions were checked by the *I*–*V* curve, and the performance was slightly lower than that obtained under H_2_ pumping conditions, reaching 200 mA·cm^–2^ at 1.4 V (Figure S3). Ethane conversion, product selectivity, and H_2_ flow generated in the reaction are displayed in Figure 8a. The EDH reaction was carried out without applying current for 60 min, giving an ethane conversion of 24%, an ethylene selectivity of 66%, and an H_2_ flow of 3.75 mL·min^–1^. Then, a current density of 300 mA·cm^–2^ was applied for 20 min to extract the H_2_ produced in the reaction, aiming to shift the thermodynamic equilibrium and improve the reaction yield. These two steps, both with and without current, were repeated once more, and the stability of the reaction performance was observed throughout the duration of the reaction. A continuous decrease in cell resistance was observed in the EIS measurements (Figure 8d,e). This fact may be attributed to the formation of coke, which can lead to an improvement in the electrical conductivity in the electrode. On the other hand, by applying a current density of 300 mA·cm^–2^, ∼13% of the H_2_ generated in the reaction could be extracted, but no change in the ethylene yield was observed. Figure 8b shows the effect of H_2_ extraction on ethane conversion, where 0% of extraction corresponds to the thermodynamic equilibrium. At equilibrium, the ethane conversion rate reached 28.45% at 650 °C without H_2_ extraction, which closely aligns with the 24% conversion observed in the experiments. However, with an H_2_ extraction of 13%, the expected improvement in conversion is only 1%. Consequently, no notable enhancement in the reaction performance was detected when H_2_ was extracted. In fact, as illustrated in Figure 8b, significant improvements in reaction performance occur only when H_2_ extraction is between 70% and 80%, particularly at temperatures of 600 and 650 °C.

Finally, after 29 h on ethane stream, a regeneration step was carried out by switching the anode gas inlet to diluted (20%) air, aiming to regenerate the electrocatalyst surface by removing the formed coke. This oxidative step was monitored by analyzing the CO_2_ formation with a GC system (Figure 8c). The cell performance was examined by using EIS measurements. A comparison of the spectra at the beginning of the electrochemical EDH experiments and after the oxidative regeneration cycle (as shown in Figure 8d,e) reveals almost no electrode degradation. This indicates that the LSCMF/BCZY_5515_-Pt/CeO_2_ electrode remains stable under the redox conditions.

**Figure 9:**
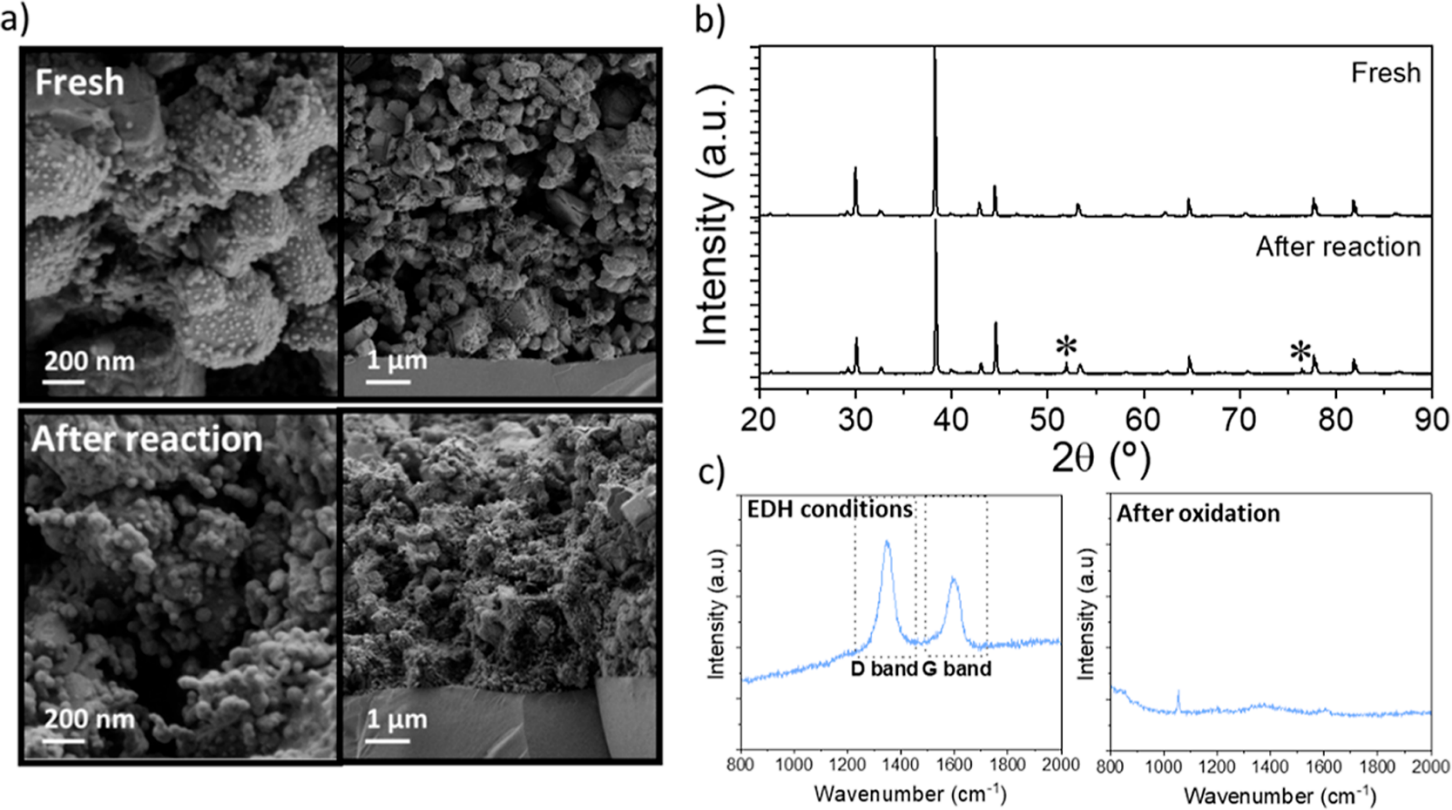
(a) SEM cross-sectional images of the cell before testing and after testing. (b) XRD patterns of the sample before and after the nonoxidative dehydrogenation of ethane reaction. (c) Raman spectra of peaks D and G of the coke generated in the reaction and after oxidation.

The postmortem cell was analyzed using SEM, XRD, and Raman spectroscopy (Figure 9) after conducting tests on the nonoxidative dehydrogenation of ethane. A comparison of the cross-sectional images of the cell interface before and after the reaction (Figure 9a) reveals an increase in the size of the Pt and CeO_2_ nanoparticles. This increase likely occurred because the cell had not been subjected to reducing conditions prior to measurement, whereas the measured cell was under reducing conditions. The literature indicates that a higher sintering of nanoparticles occurs in reducing environments.^30^ Furthermore, the anode after operation does not exhibit degradation and shows good dispersion of the catalytic nanoparticles. Figure 9b compares the XRD patterns of the cells, and no degradation was observed as no secondary phases were detected. The only noticeable difference is the peak marked with an asterisk, which corresponds to nickel. This occurs because, after the reaction, nickel oxide in the cathode is reduced. Finally, the sample was characterized using Raman spectroscopy to examine the coke formed during the reaction and after exposure to coke oxidation conditions (Figure 9c). Under the reaction conditions, coke formation is observed, with characteristic peaks indicative of coke visible in the Raman spectrum. The D-band peak appears at 1346.6 cm^–1^ with an intensity of 2033.13 (a.u.), while the G-band peak is observed at 1589.9 cm^–1^ with an intensity of 1239.21 (a.u.). The *I*_D_/*I*_G_ ratio is 1.64, indicating that the coke formed during the reaction is mainly composed of amorphous carbon. A higher *I*_D_/*I*_G_ ratio signifies a greater level of structural disorder and defects.^31,32^Figure 9c clearly shows the removal of coke following the regeneration treatment.

## 4 Conclusions

In this work, the chemical compatibility of five different perovskite materials (La_0.8_Sr_0.2_Cr_0.25_Mn_0.75_O_3−δ_ (LSCM_75_), La_0.7_Sr_0.1_Ce_0.2_Cr_0.5_Mn_0.5_O_3−δ_ (LSCeCM), La_0.7_Sr_0.1_Nd_0.2_Cr_0.5_Mn_0.5_O_3−δ_ (LSNCM), La_0.8_Sr_0.2_Cr_0.5_Fe_0.5_O_3−δ_ (LSCF), and La_0.8_Sr_0.2_Cr_0.5_Mn_0.25_Fe_0.25_O_3−δ_ (LSCMF) with the protonic electrolyte material BaCe_0.55_Zr_0.3_Y_0.15_O_3−δ_ (BCZY_5515_) was studied. The microstructural and electrochemical properties of the developed electrodes revealed that in humidified H_2_, surface reactions limit the electrochemical performance. Further improvement in the electrochemical performance was achieved by infiltrating Pt/CeO_2_ nanoparticles under reducing atmospheres. The best electrochemical performance was observed for the LSCMF/BCZY_5515_ electrode infiltrated with binary Pt/CeO_2_, resulting in a reduction of the polarization resistance by more than 1 order of magnitude at 700 °C. Given the promising results, we performed consecutive redox cycles (humidified air–humidified C_2_H_6_–H_2_), which demonstrated the redox stability of the electrode and suitability for the dehydrogenation of ethane.

Therefore, the nonoxidative dehydrogenation of ethane test was carried out using an electrochemical cell consisting of (a) an anode made of LSCMF/BCZY_5515_ infiltrated with Pt/CeO_2_ and (b) a thin BaZr_0.44_Ce_0.36_Y_0.2_O_3−δ_ electrolyte supported on (c) a Ni–SrZr_0.5_Ce_0.4_Y_0.1_O_2.95_ cathode. The reaction performance remained stable throughout the test; however, a gradual decrease in cell resistance was observed in the EIS measurements, which was attributed to coke formation. The accumulated coke was successfully removed through an oxidative regeneration cycle, with no degradation observed afterward.

These results demonstrate that the LSCMF/BCZY_5515_-Pt/CeO_2_ electrode is stable under the redox conditions. Postmortem analysis of the cell using SEM, XRD, and Raman spectroscopy revealed no signs of degradation. Based on these findings, it can be concluded that the LSCMF/BCZY_5515_ electrode infiltrated with Pt/CeO_2_ nanoparticles presents excellent electrochemical performance and is a promising redox-stable anode candidate for electrochemically promoted catalytic hydrocarbon conversion reactions, particularly when coke is produced, and must be removed through oxidative regeneration cycles.

## Supporting Information Available

Nyquist and Bode plots, *I*–*V* curve under nonoxidative dehydrogenation of ethane reaction conditions, and fitted parameters from EIS analysis (PDF)
