# Supplementary material for: Redox-Stable Electrodes for Ethane Dehydrogenation Based on Proton Ceramic Electrochemical Reactors
Source: ACS Appl Energy Mater. 2025 Mar 27;8(7):4345–54. doi: 10.1021/acsaem.4c03281 (PMC12001183; doi:10.1021/acsaem.4c03281)
Supplement: Supplementary file 1 — ae4c03281_si_001.pdf [file ae4c03281_si_001.pdf]

Supporting Information

Redox-stable electrodes for ethane dehydrogenation based on proton ceramic electrochemical reactors.

Elena Barrio-Querol[1], Laura Almar[1], David Catalán-Martínez[1], Kwati Leonard[2], José Manuel Serra[1,*], Sonia Escolástico[1,*]

[1]*Instituto de Tecnología Química (Universitat Politècnica de València-Consejo Superior de Investigaciones Científicas), 46022 València, Spain*

[2] *Center for Energy Systems Design (CESD), International Institute for Carbon-Neutral Energy Research (I2CNER), Kyushu University, 744 Motooka, Nishi-ku, Fukuoka, 819-0395, Japan*

**jmserra@itq.upv.es*

**soesro@itq.upv.es*

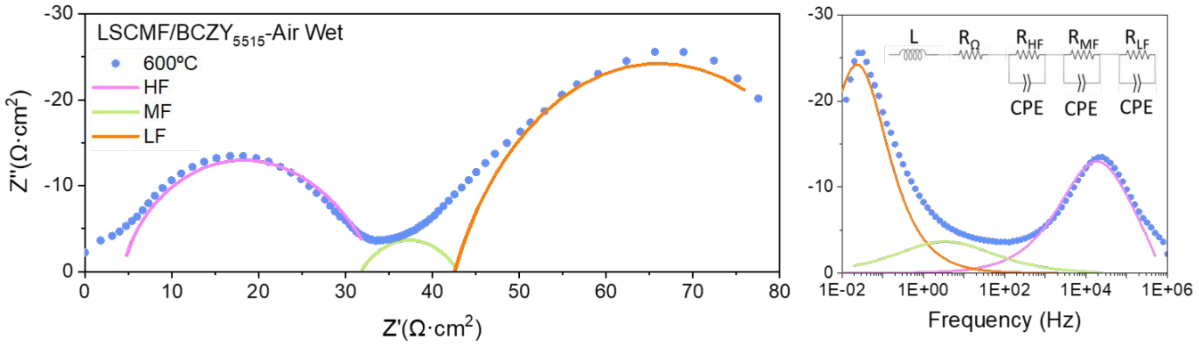

Figure S1. Nyquist (a) and Bode (b) plots for the electrode LSCMF/BCZY$_{5515}$ measured at 600 °C under humidified air. Ohmic resistance has been subtracted for clarity reasons.

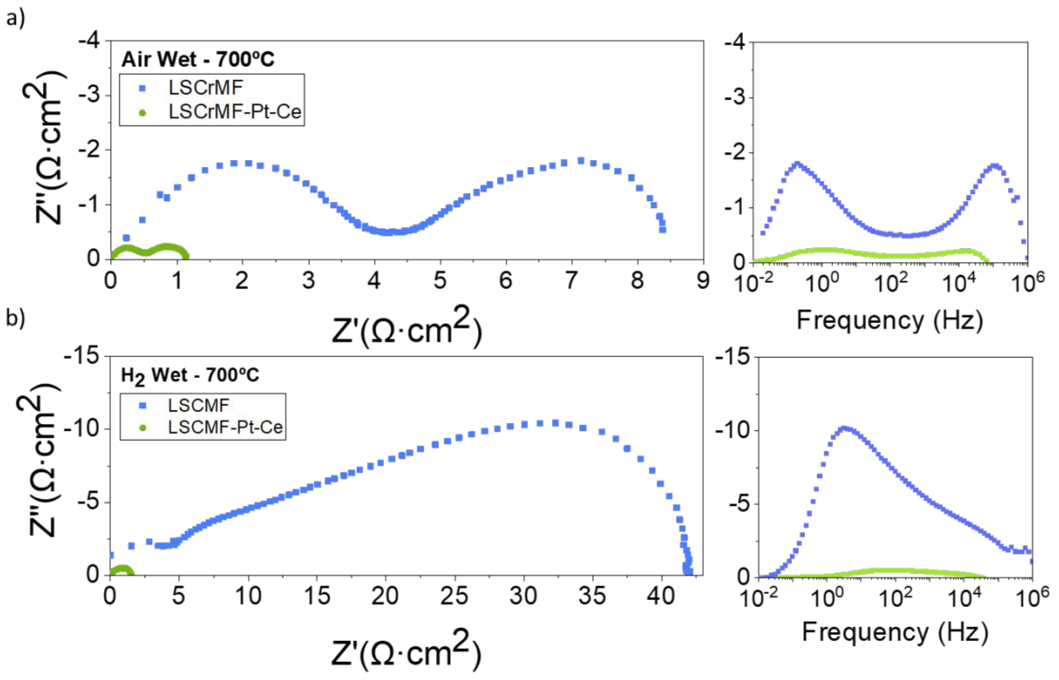

Figure S2. Impedance spectra for the symmetrical cell of LSCMF/BCZY$_{5515}$ electrode without and with infiltration of Pt/CeO$_2$ at 700 °C in 3% H$_2$O saturated synthetic air (a) and 10% of H$_2$ in Ar (b).

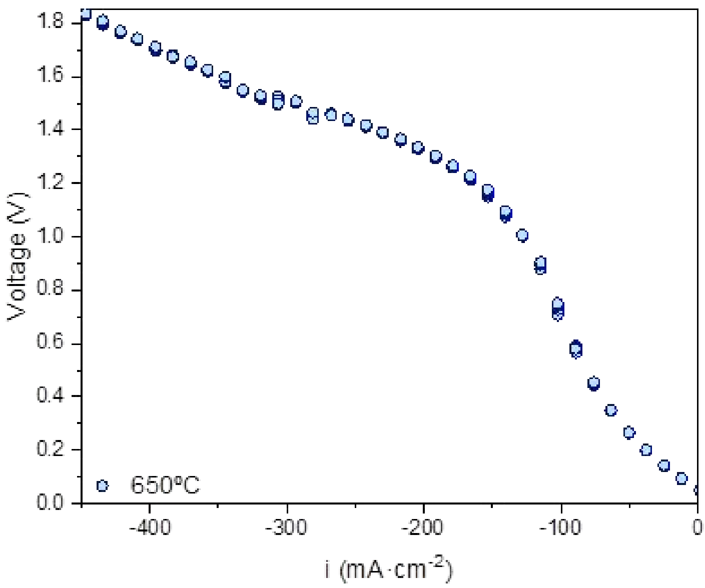

Figure S3. i-V curve under non-oxidative dehydrogenation of ethane reaction conditions at 650 °C.

1. Fittings EIS

Impedance spectra were fitted with the equivalent circuit shown in the inset of Figure S1 where HF, MF and LF correspond to the high, medium, and low associated frequencies. The following tables show the obtained results.

Table S1. The fitted parameters of the equivalent circuit for the sample LSCMF/BCZY$_{5515}$.

T (°C)	Gas	R_Ω ($\Omega\cdot cm^{-2}$)	R_{LF} ($\Omega\cdot cm^{-2}$)	Ceq_{LF} ($F\cdot cm^{-2}$)	f_{LF} (Hz)	R_{MF} ($\Omega\cdot cm^{-2}$)	Ceq_{MF} ($F\cdot cm^{-2}$)	f_{MF} (Hz)	R_{HF} ($\Omega\cdot cm^{-2}$)	Ceq_{HF} ($F\cdot cm^{-2}$)	f_{HF} (Hz)
600	Air	20.31	24.85	0.14	0.02	5.21	$2.42\cdot10^{-3}$	6.29	14.94	$2.92\cdot10^{-7}$	18267.39
700	Air	9.80	1.67	0.40	0.12	0.68	$1.12\cdot10^{-2}$	10.38	2.75	$2.55\cdot10^{-7}$	113803.84
800	Air	6.65	0.20	0.25	1.61	0.10	$4.15\cdot10^{-4}$	1889.62	0.18	$7.38\cdot10^{-6}$	59461.28
600	H_2	42.53	29.17	$2.35\cdot10^{-3}$	1.16	18.02	$5.10\cdot10^{-5}$	86.59	15.04	$5.38\cdot10^{-7}$	9830.27
700	H_2	23.47	5.90	$6.19\cdot10^{-3}$	2.18	9.03	$1.87\cdot10^{-4}$	47.22	4.79	$1.40\cdot10^{-6}$	11854.74
800	H_2	13.91	2.55	$4.98\cdot10^{-3}$	6.27	2.65	$6.49\cdot10^{-4}$	46.24	1.78	$1.80\cdot10^{-5}$	2477.75

Table S2. The fitted parameters of the equivalent circuit for the sample LSNCM/BCZY$_{5515}$.

T (°C)	Gas	R_Ω ($\Omega\cdot cm^{-2}$)	R_{LF} ($\Omega\cdot cm^{-2}$)	Ceq_{LF} ($F\cdot cm^{-2}$)	f_{LF} (Hz)	R_{MF} ($\Omega\cdot cm^{-2}$)	Ceq_{MF} ($F\cdot cm^{-2}$)	f_{MF} (Hz)	R_{HF} ($\Omega\cdot cm^{-2}$)	Ceq_{HF} ($F\cdot cm^{-2}$)	f_{HF} (Hz)
600	Air	13.42	11.87	0.12	0.05	5.98	$1.58\cdot10^{-3}$	8.43	18.68	$1.33\cdot10^{-7}$	32074.91
700	Air	6.31	0.70	0.27	0.43	0.43	$2.11\cdot10^{-3}$	87.86	2.75	$7.22\cdot10^{-5}$	400.87
800	Air	4.61	0.09	0.16	5.21	0.07	$3.35\cdot10^{-4}$	3592.94	0.17	$6.56\cdot10^{-6}$	71310.75
600	H_2	11.41	56.11	0.06	0.02	5.62	$2.46\cdot10^{-4}$	57.49	4.49	$2.67\cdot10^{-6}$	6638.69
700	H_2	7.35	5.29	0.03	0.56	2.02	$9.36\cdot10^{-4}$	42.02	1.42	$7.22\cdot10^{-5}$	777.26
800	H_2	5.82	0.92	0.03	3.39	0.82	$3.06\cdot10^{-3}$	31.84	0.62	$3.14\cdot10^{-5}$	4071.55

Table S3. The fitted parameters of the equivalent circuit for the sample LSCF/BCZY$_{5515}$.

T (°C)	Gas	R_Ω ($\Omega\cdot cm^{-2}$)	R_{LF} ($\Omega\cdot cm^{-2}$)	Ceq_{LF} ($F\cdot cm^{-2}$)	f_{LF} (Hz)	R_{MF} ($\Omega\cdot cm^{-2}$)	Ceq_{MF} ($F\cdot cm^{-2}$)	f_{MF} (Hz)	R_{HF} ($\Omega\cdot cm^{-2}$)	Ceq_{HF} ($F\cdot cm^{-2}$)	f_{HF} (Hz)
600	Air	8.95	6.06	0.21	0.06	2.58	0.01	2.78	2.75	$1.65\cdot10^{-6}$	17585.60
700	Air	4.85	0.50	0.29	0.56	0.21	$2.51\cdot10^{-3}$	148.46	0.48	$2.84\cdot10^{-6}$	58436.04
800	Air	2.74	0.05	0.35	4.51	0.04	$1.07\cdot10^{-3}$	1742.62	0.16	$1.66\cdot10^{-6}$	297398.33
600	H_2	8.55	42.11	0.04	0.04	9.23	$1.15\cdot10^{-3}$	7.52	4.51	$1.81\cdot10^{-5}$	976.60
700	H_2	6.39	3.60	0.04	0.56	3.81	$4.22\cdot10^{-3}$	4.95	1.87	$4.33\cdot10^{-5}$	986.00
800	H_2	4.86	0.76	0.04	2.56	0.95	$6.43\cdot10^{-3}$	13.06	0.88	$1.11\cdot10^{-4}$	808.58

Table S4. The fitted parameters of the equivalent circuit for the sample LSCM$_{75}$/BCZY$_{5515}$.

T (°C)	Gas	R_{Ω} ($\Omega\cdot cm^{-2}$)	R_{LF} ($\Omega\cdot cm^{-2}$)	Ceq_{LF} ($F\cdot cm^{-2}$)	f_{LF} (Hz)	R_{MF} ($\Omega\cdot cm^{-2}$)	Ceq_{MF} ($F\cdot cm^{-2}$)	f_{MF} (Hz)	R_{HF} ($\Omega\cdot cm^{-2}$)	Ceq_{HF} ($F\cdot cm^{-2}$)	f_{HF} (Hz)
600	Air	20.60	25.07	0.14	0.02	5.17	0.14	193.72	14.73	$3.02\cdot10^{-7}$	17902.46
700	Air	10.22	1.67	0.35	0.06	0.68	0.35	88.16	2.75	$3.21\cdot10^{-8}$	308576.00
800	Air	9.15	0.45	0.23	0.78	0.21	0.23	1.64	0.89	$2.10\cdot10^{-7}$	425525.52
600	H_2	27.88	17.57	0.11	0.28	27.04	$9.58\cdot10^{-4}$	3083.21	20.45	$2.49\cdot10^{-7}$	15653.90
700	H_2	21.71	6.25	0.02	0.71	4.65	$1.33\cdot10^{-3}$	24667.55	6.97	$2.09\cdot10^{-7}$	54534.23
800	H_2	15.50	2.69	$6.21\cdot10^{-3}$	4.77	1.75	$1.70\cdot10^{-3}$	332648.09	2.76	$6.72\cdot10^{-8}$	429112.32

Table S5. The fitted parameters of the equivalent circuit for the sample LSCeCM/BCZY$_{5515}$.

T (°C)	Gas	R_{Ω} ($\Omega\cdot cm^{-2}$)	R_{LF} ($\Omega\cdot cm^{-2}$)	Ceq_{LF} ($F\cdot cm^{-2}$)	f_{LF} (Hz)	R_{MF} ($\Omega\cdot cm^{-2}$)	Ceq_{MF} ($F\cdot cm^{-2}$)	f_{MF} (Hz)	R_{HF} ($\Omega\cdot cm^{-2}$)	Ceq_{HF} ($F\cdot cm^{-2}$)	f_{HF} (Hz)
600	Air	-	-	-	-	-	-	-	-	-	-
700	Air	1079	252.37	$1.97\cdot10^{-5}$	16.04	99.94	$1.08\cdot10^{-8}$	$7.40E\cdot10^{4}$	41.92	$7.27\cdot10^{-9}$	$2.61\cdot10^{5}$
800	Air	16.49	0.20	0.03	13.61	0.21	$3.92\cdot10^{-8}$	$9.81\cdot10^{6}$	2.57	$3.76\cdot10^{-8}$	$8.25\cdot10^{5}$
600	H_2	429.8	3020.55	$3.88\cdot10^{-4}$	0.07	806.67	$1.69\cdot10^{-6}$	58.4	492.72	$6.86\cdot10^{-9}$	$2.35\cdot10^{4}$
700	H_2	208.2	219.64	$3.37\cdot10^{-4}$	1.08	144.41	$4.25\cdot10^{-6}$	130	101.44	$1.96\cdot10^{-8}$	$4.01\cdot10^{4}$
800	H_2	172	102.36	$2.91\cdot10^{-4}$	5.00	87.73	$2.30\cdot10^{-5}$	39.4	5.65	$1.82\cdot10^{-7}$	$7.73\cdot10^{4}$

Table S6. The fitted parameters of the equivalent circuit for the sample LSCMF/BCZY$_{5515}$ infiltrated with Pt/CeO_2.

T (°C)	Gas	R_{Ω} ($\Omega\cdot cm^{-2}$)	R_{LF} ($\Omega\cdot cm^{-2}$)	Ceq_{LF} ($F\cdot cm^{-2}$)	f_{LF} (Hz)	R_{MF} ($\Omega\cdot cm^{-2}$)	Ceq_{MF} ($F\cdot cm^{-2}$)	f_{MF} (Hz)	R_{HF} ($\Omega\cdot cm^{-2}$)	Ceq_{HF} ($F\cdot cm^{-2}$)	f_{HF} (Hz)
600	Air	6.90	2.76	0.14	1.92	1.08	$5.76\cdot10^{-4}$	127.81	2.32	$5.75\cdot10^{-7}$	$1.28\cdot10^{5}$
700	Air	4.42	0.29	0.24	1.13	0.13	$7.09\cdot10^{-4}$	849.69	0.27	$6.98\cdot10^{-6}$	$4.21\cdot10^{4}$
800	Air	2.56	0.03	0.86	2.89	0.02	$5.40\cdot10^{-2}$	65.53	0.01	$1.73\cdot10^{-3}$	$4.79\cdot10^{3}$
600	H_2	7.79	0.72	0.02	5.41	1.36	$3.52\cdot10^{-4}$	165.71	0.61	7.45E-06	$1.74\cdot10^{4}$
700	H_2	5.19	0.24	0.02	16.31	0.32	$1.01\cdot10^{-3}$	247.92	0.25	1.72E-05	$1.89\cdot10^{4}$
800	H_2	4.09	0.07	0.03	33.96	0.25	$2.87\cdot10^{-3}$	111.65	0.12	1.55E-04	$4.21\cdot10^{3}$

Table S7. The fitted parameters of the equivalent circuit for the sample LSCF/BCZY$_{5515}$ infiltrated with Pt/CeO_2.

T (°C)	Gas	R_{Ω} ($\Omega\cdot cm^{-2}$)	R_{LF} ($\Omega\cdot cm^{-2}$)	Ceq_{LF} ($F\cdot cm^{-2}$)	f_{LF} (Hz)	R_{MF} ($\Omega\cdot cm^{-2}$)	Ceq_{MF} ($F\cdot cm^{-2}$)	f_{MF} (Hz)	R_{HF} ($\Omega\cdot cm^{-2}$)	Ceq_{HF} ($F\cdot cm^{-2}$)	f_{HF} (Hz)
600	Air	11.45	30.05	0.03	0.08	22.72	$2.87\cdot10^{-5}$	122.19	40.40	$2.39\cdot10^{-8}$	$8.25\cdot10^{4}$
700	Air	6.22	1.34	0.12	0.51	4.10	$1.65\cdot10^{-6}$	1.17E+04	3.03	$5.67\cdot10^{-8}$	$4.63\cdot10^{5}$
800	Air	3.49	0.09	0.41	2.27	1.18	$9.42\cdot10^{-7}$	7.16E+04	0.71	$4.33\cdot10^{-7}$	$2.60\cdot10^{6}$
600	H_2	8.21	0.96	0.01	6.31	2.40	$1.91\cdot10^{-4}$	173.70	1.55	$1.82\cdot10^{-6}$	$2.81\cdot10^{4}$
700	H_2	6.02	0.40	0.02	10.35	0.61	$6.67\cdot10^{-4}$	197.22	0.40	$5.84\cdot10^{-6}$	$3.40\cdot10^{4}$
800	H_2	4.60	0.26	0.01	21.25	0.19	$1.94\cdot10^{-3}$	220.36	0.14	$1.20\cdot10^{-4}$	$4.87\cdot10^{3}$

Table S8. The fitted parameters of the equivalent circuit for the sample LSNCM/BCZY$_{5515}$ infiltrated with Pt/CeO_2.

T (°C)	Gas	R_{Ω} ($\Omega\cdot cm^{-2}$)	R_{LF} ($\Omega\cdot cm^{-2}$)	Ceq_{LF} ($F\cdot cm^{-2}$)	f_{LF} (Hz)	R_{MF} ($\Omega\cdot cm^{-2}$)	Ceq_{MF} ($F\cdot cm^{-2}$)	f_{MF} (Hz)	R_{HF} ($\Omega\cdot cm^{-2}$)	Ceq_{HF} ($F\cdot cm^{-2}$)	f_{HF} (Hz)
600	Air	13.51	3.03	0.22	0.12	2.73	$4.54\cdot10^{-4}$	64.27	3.31	$4.12\cdot10^{-7}$	$5.82\cdot10^{4}$
700	Air	10.15	0.37	0.32	0.66	0.29	$1.43\cdot10^{-4}$	$1.93\cdot10^{3}$	0.65	$2.15\cdot10^{-6}$	$5.71\cdot10^{4}$
800	Air	4.75	0.07	0.37	3.15	0.08	$1.98\cdot10^{-4}$	$5.24\cdot10^{3}$	0.05	$7.66\cdot10^{-5}$	$2.11\cdot10^{4}$
600	H_2	15.61	2.21	0.01	3.48	1.07	$3.85\cdot10^{-4}$	193.96	0.66	$4.87\cdot10^{-6}$	$2.46\cdot10^{4}$
700	H_2	7.09	0.89	0.01	12.93	0.38	$2.62\cdot10^{-4}$	791.07	0.12	$1.78\cdot10^{-5}$	$3.59\cdot10^{4}$
800	H_2	5.28	0.14	1.03	0.57	0.27	$2.07\cdot10^{-4}$	1447.32	0.29	$7.71\cdot10^{-3}$	35.88
